# Protection of calcium silicate/sodium phosphate/fluoride toothpaste with serum on enamel and dentin erosive wear

**DOI:** 10.1590/1678-7757-2021-0081

**Published:** 2021-10-01

**Authors:** Rayssa Ferreira ZANATTA, Daniele Mara da Silva ÁVILA, Morgana Menezes MAIA, Ítallo Emídio Lira VIANA, Tais SCARAMUCCI, Carlos Rocha Gomes TORRES, Alessandra Bühler BORGES

**Affiliations:** 1 Universidade de Taubaté Departamento de Odontologia Restauradora TaubatéSão Paulo Brasil Universidade de Taubaté - UNITAU, Departamento de Odontologia Restauradora, Taubaté, São Paulo, Brasil.; 2 Universidade Estadual Paulista Instituto de Ciência e Tecnologia de São José dos Campos Departamento de Odontologia Restauradora São Paulo Brasil Universidade Estadual Paulista – UNESP, Instituto de Ciência e Tecnologia de São José dos Campos, Departamento de Odontologia Restauradora, São Paulo, Brasil.; 3 Universidade de São Paulo Faculdade de Odontologia Departamento de Odontologia Restauradora São Paulo Brasil Universidade de São Paulo, Faculdade de Odontologia - FOUSP, Departamento de Odontologia Restauradora, São Paulo, Brasil.

**Keywords:** Fluoride, Enamel, Dentin, Tooth erosion, Toothpaste

## Abstract

**Objective:**

To evaluate the effect of a toothpaste containing calcium silicate/sodium phosphate/fluoride associated or not to the boost serum (BS) against erosive tooth wear (ETW) on enamel and dentin.

**Methodology:**

Bovine enamel and dentin specimens were subjected to an erosion-abrasion cycling model (1% citric acid - pH 3.6 -2 min / artificial saliva – 60 min, 4×/day, 5 days). Toothbrushing was performed for 15 s (2 min exposed to slurry), 2×/day, with the toothpastes (n=10): control without fluoride (Weleda), Arg/Ca/MFP (Colgate Pro-Relief), Si/PO_4_/MFP (Regenerate-Unilever), and Si/PO_4_/MFP/BS (Si/PO_4_/MFP with dual BS – Advanced Enamel Serum-Unilever). The effect of treatments on the eroded tissues was assessed by surface microhardness in the first day, and surface loss (SL) resulting from ETW was evaluated by profilometry (μm) after three and five days. Additional dentin specimens (n=5/group) were subjected to 20,000 brushing cycles to verify the abrasivity of the toothpastes. Data were subjected to ANOVA and correlation tests (5%).

**Results:**

For enamel, no difference in microhardness was observed among the treated groups, and similar SL was obtained after 5 days. For dentin, Si/PO_4_/MFP/BS resulted in higher microhardness values, but none of the groups presented significantly lower SL than the control. There was no significant correlation between SL and abrasiveness.

**Conclusion:**

The calcium silicate/sodium phosphate toothpaste and serum increased microhardness of eroded dentin, but they did not significantly reduce enamel and dentin loss compared to the non-fluoride control toothpaste. The abrasiveness of the toothpastes could not predict their effect on ETW.

## Introduction

The initial stage of enamel dissolution (early erosion) is associated to the weakening of the surface when it contacts an acidic solution. This softened layer presents reduced hardness and is more prone to abrasive wear.^1^ The maintenance of the erosive/abrasive events induces to the more advanced stages of the process, with loss of the dental hard tissues, which is known as erosive tooth wear (ETW).^2^ This is an increasing condition, which affects populations worldwide, especially children, adolescents, and young adults.^3,4^ It has been associated with high consumption of acidic foodstuff, changes in lifestyles and some medical conditions, such as gastroesophageal reflux and frequent vomiting.^3,5^ Although the main etiological factors associated with ETW are known, controlling the exposure to these factors is challenging, because it involves the individual’s compliance.^6^ Thus, different strategies have been investigated to prevent and control ETW. The use of conventional fluoride toothpastes has shown some protection, although it seems limited.^7-10^ Noteworthy, even with the wide use of these products, the global prevalence of erosive tooth wear is high, being estimated in 20-45% in permanent teeth.^5^ Therefore, agents intended to increase the protective potential of toothpastes against ETW are relevant.

Some agents added to the toothpastes may modulate their anti-erosive effect on enamel (presence of Sn^2^, higher concentration of Ca^2^ and PO_4_-)^11^ and dentin (concentration of F-).^12^ Furthermore, dentifrices containing desensitizing agents, such as arginine/calcium carbonate, associated with fluoride were found to protect enamel against erosive attacks *in vitro* and *in situ*,^13^ whereas for dentin, the evidence is still scarce. Also, there are some previous investigations about the protective effect of different toothpastes against ETW, but the evidence is not robust enough.^7,10^ Another aspect that should be considered is that the dentifrices are used during brushing, thus the abrasive potential is an important factor that can influence their protection against ETW.^7^

A dentifrice containing calcium silicate and sodium phosphate salts (monosodium phosphate and trisodium phosphate) with 1,450 ppm of sodium monofluorophosphate presented promising *in vitro* and *in situ* results regarding the control of initial erosion on enamel, because it lead to the rehardening of the softening layer.^14,15^ Furthermore, a dual-phase boost serum (BS) gel containing calcium silicate salts and sodium phosphate plus sodium fluoride was developed to complement the dentifrice action in the treatment of early erosive lesions.^15^ Their mechanism of action is based on the deposition of calcium silicate over the enamel surface, protecting it from demineralization, whereas the dual-phase gel acts by promoting the remineralization of eroded enamel.^14-17^ However, the ability of this system (dentifrice and boost serum gel) to control the ETW on enamel is not fully established, with variable results regarding efficacy, especially when abrasion is present in the model.^18-21^ Moreover, there is not much data about the effect of these products on ETW in dentin.

Thus, our study aimed to evaluate the effect of the toothpaste containing calcium silicate/sodium phosphate and fluoride, associated or not to the dual phase boost serum (BS) against erosive wear of enamel and dentin. The null hypotheses tested were; 1. There is no difference among the microhardness of eroded tissues treated with the tested products; 2. For both substrates, surface loss is not different among groups after 3 and 5-days erosion-abrasion cycling; 3. There is no significant correlation between dentin surface loss after cycling and dentin abrasiveness.

## Methodology

### Study Design

Enamel and dentin polished specimens, obtained from bovine incisors (n=10 / group), were exposed to 5 days of an erosion-abrasion cycling. Four different treatments were tested; dentifrice without fluoride; dentifrice with arginine, calcium carbonate and sodium monofluorophosphate; dentifrice with calcium silicate, sodium phosphate and sodium monofluorophosphate; and the association of the previous one with dual phase boost serum gel, containing calcium silicate/sodium phosphate/sodium monofluorophosphate and sodium fluoride. The variables were surface microhardness (SMH), measured at baseline (B) and at the first day of cycling, after first acid challenge (E) and treatment (T), and surface loss (SL) measured by contact profilometry after the 3^rd^ and 5^th^ days of cycling. Furthermore, the abrasiveness of the dentifrices was assessed by profilometry after 5, 10, 15 and 20 thousand toothbrushing cycles on dentin.

### Sample Preparation

Freshly extracted and intact bovine incisors were selected, cleaned, and stored in 0.1% thymol solution at 4ºC, until required. Crowns were separated from roots using a diamond disk, and one hundred cylindrical specimens were obtained from their labial surface using a custom-made diamond trephine mill with 3 mm internal diameter.^22^ Specimens were ground flat with water-cooled silicon carbide (SiC) paper discs (#1200 / Fepa-P, Struers, Ballerup, Denmark) to standardize a height in 2 mm with the aid of a metallic device, and then allocated into two groups (n=50) according with tooth substrate (enamel or root dentin).

Specimens were embedded in acrylic resin (ExtecFast Cure Acrylic, ExtecCorp, Enfield, CT, USA) using a silicon mold and, after cure, were polished using sequential aluminum oxide abrasive papers: 1200-, 2400- and 4000-grit (FEPA-P, Struers, Ballerup, Denmark) under water irrigation, for 30, 60 and 120 s, respectively. After each paper grit change, specimens were kept in ultrasonic bath for 10 minutes to remove debris and abrasive grains. Then, they were examined in stereomicroscope (Carl Zeiss – Stemi 2000 -20×) to ensure the absence of cracks or other surface defects.

### Microhardness

Initial Knoop surface microhardness (SMH_B_) was determined with 50 g load during 10 s for enamel, and with 10 g during 10 s for dentin. Three measurements with 100 μm of distance between them were performed in each specimen and averaged. Specimens presenting a microhardness variation higher than 15% of mean value were replaced.

### Experimental groups

Considering initial microhardness measurements, enamel and dentin specimens were separately stratified in four groups (n=10) according to the treatment; Control – dentifrice without fluoride (negative control – Weleda Sole Zahncreme, Weleda); Arg/Ca/MFP – arginine (8%), calcium carbonate and sodium monofluorophosphate dentifrice (Colgate Pro-Relief); Si/PO_4_/MFP – calcium silicate, sodium phosphate and sodium monofluorophosphate (1450ppm F^-^) (Regenerate, Unilever); Si/ PO_4_/MFP/BS – Si/PO_4_/MFP dentifrice associated with a dual phase gel (Boost Serum), comprising two parts, A – calcium silicate/sodium phosphate/sodium monofluorophosphate (1450ppm F^-^) and B – 1450 ppm F^-^ sodium fluoride (Regenerate system + Advanced Enamel Boost Serum - Unilever). The composition and pH values of all dentifrices tested are shown in Table 1.

**Table 1:** The composition of the dentifrices and pH-values for the slurries

Group	Dentifrice	Composition	pH

Control	Weleda (Weleda – Arlesheim, Switzerland)	Sodium Bicarbonate, Water, Glycerin, Silica, Peppermint, Sodium Chloride, Commiphora Myrrha Resin Extract, Krameria Triandra Root Extract, Guar.	8.07
Arg/Ca/MFP	Colgate Sensitive Pro-Relief (Colgate Palmolive, Brazil)	Water, Sorbitol, Sodium Lauryl Sulfate, Aroma, Cellulose Gum, Sodium Bicarbonate, Tetrasodium pyrophosphate, Sodium Saccharin, Benzyl alcohol, Xantam gum, Limonene, Sodium Monofluorophosphate (1450 ppm), Arginine/Calcium Carbonate.	8.74
Si/PO4/MFP	Regenerate (Unilever, France)	Water, Glycerin, Calcium Silicate, PEG 8, Hydrated Silica, Trisodium Phosphate, Sodium Phosphate, PEG-60, Sodium Lauryl Sulfate, Sodium Monofluorophosphate (1450 ppm), flavor, Synthetic Fluorphlogopite, Sodium Saccharin, Polyacrylic Acid, Tin Oxide, Limonene.	8.78
Si/PO4/MFP/BS	Regenerate Advanced Enamel Boost Serum (Unilever, France)	A: Water, Glycerin, Calcium Silicate, PEG 8, Hydrated Silica, Trisodium Phosphate, Sodium Phosphate, PEG-60, Sodium Lauryl Sulfate, Sodium Monofluorophosphate (1450 ppm), flavour, Synthetic Fluorphlogopite, Sodium Saccharin, Polyacrylic Acid, Tin Oxide, Limonene.	/
		B: Water, Glycerin, Cellulose Gum, Sodium Fluoride, Benzyl Alcohol, Ethylhexylglycerin, Phenoxyethanol, Sodium Fluoride (1450 ppm).	/

### Profilometry

To maintain the reference surfaces for lesion-depth determination (profilometry) and to allow exact replacement, two parallel grooves were marked on the sides of the acrylic resin surface to serve as guides. Before the erosive-abrasive challenge, profiles of each specimen were obtained from the enamel and dentin surfaces with a contact profilometer (MaxSurf XT 20, Mahr, Goettingen, Germany). The diamond stylus moved from the first reference area in acrylic resin into the second one (4.2 mm long). Three profile measurements were performed for each specimen at intervals of 0.25 mm.

### Erosion-abrasion challenge

**Figure 1:**
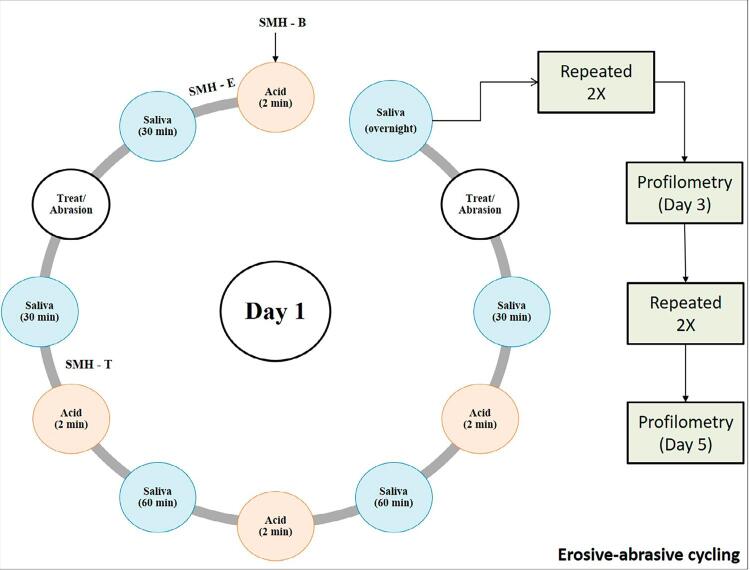
Daily erosive-abrasive challenges. SHM refers to the surface microhardness measurements. This was repeated for 5 days, and profilometric assay was conducted by the end of the 3rd and 5th days

An erosion-abrasion cycling model was performed for 5 days. The daily treatment consisted of immersing specimens in 1% citric acid (2 min - 4 times / day, pH adjusted to 3.6 with KOH)^23^, followed by immersion in artificial saliva (6 times / day) for 30 minutes before treatments and 60 minutes between exposure to citric acid. Abrasion plus immersion in the toothpaste slurry was performed twice a day simulating two daily brushings. Figure 1 shows a chart of the erosive/abrasive cycling.

Toothpaste slurries were prepared immediately before each treatment (1:3 – dentifrice : artificial saliva), to use fresh solutions on the specimens.^15^ Artificial saliva used in our study was composed by 0.002 g of ascorbic acid, 0.030 g of glucose, 0.580 g of sodium chloride (NaCl), 0.170 g of calcium chloride (CaCl_2_), 0.160 g of ammonium chloride (NH_4_Cl), 1.270 g of potassium chloride (KCl), 0.160 g sodium tiocianate (NaSCN), 0.330 g monobasic potassium phosphate (KH_2_PO_4_), 0.200 g of urea and 0.340 g di-sodium phosphate (Na_2_HPO_4_) in 1000 mL of distilled water.^24^ For the Si/PO_4_/MFP/BS group, the boost serum was weighed (part A and part B) in the proportion 1:1 and applied on enamel and dentin surfaces after the second abrasion challenge for 3 min, 1×/day for 3 days, following manufacturer’s instructions.

Abrasion was performed using an automatic brushing equipment (MEV-2T – Odeme Dental Research, Luzerna, SC, Brazil). Standard toothbrushes (Sanifill Ultra Professional 39, São Paulo, Brazil) were adapted in the brushing machine, angled 12° in relation to the specimen surface to minimize grooves formation. During brushing, the right and left sides of the specimens, corresponding to acrylic resin with the reference groves, were protected with a opened window of 2-mm wide stainless-steel mask (0.1-mm thick), leaving an exposed area in the center of the specimen and preventing the abrasion of reference areas for the profilometric analysis. During the abrasive challenge, the specimens were immersed in the slurry for 120 seconds (15 s of brushing – 2 strokes/s, 200 g load, followed by 105 s without brushing).^25^ Between cycling days, the specimens were stored overnight in 100% relative humidity at 4ºC.

### Final microhardness and profilometry

Microhardness analysis was used to check the effect of the treatments on eroded tissues in the first day of the cycle. Measurements were performed in three moments (figure 1): B – baseline; E – after first acid, and T – after the treatment and immersion in artificial saliva. The microhardness parameters used were the same as described for the initial measurements, and its alteration was calculated in terms of percentage using the respective formula: $\%{SMH}_{\text{alt }}=|{SMH}_{T}/{SMH}_{E})*100$.

Final profiles were obtained at the end of the 3^rd^ and the 5^th^ days of the erosive-abrasive cycle, and performed with the same parameters of the initial profiles. Dentin profiles were obtained in moistened conditions. Surface loss data were estimated by the height difference between initial and final profiles using profilometer software (Mahr Surf XCR 20 4.50-07 SP3, 2011).

### Toothpastes abrasivity analysis

To check the differences in the abrasiveness of the toothpastes used in this study, additional dentin specimens were prepared (n=5, each group) as previous described and subjected to 20,000 abrasion cycles. Profilometry was assessed 5 times (initial, after 5,000, 10,000, 15,000 and 20,000 abrasion cycles) to create the surface loss pattern of each dentifrice.

### Statistical analysis

Data were checked for normality and homogeneity assumptions (Kolmogorov Smirnov and Levene tests), and then one-way ANOVA test was applied for microhardness and profilometry values, separated for enamel and dentin data, followed by Tukey’s test. Dentin loss data after 20,000 abrasive cycles was analyzed by one-way ANOVA, followed by Tukey’s test. The correlation between surface loss data at the end of the cycling and dentin abrasiveness was made using the Pearson’s correlation test. Statistica for Windows Software (StatSoft, Tulsa, OK, USA) was used and a 5% level of significance was considered in all the analyses.

## Results

### Microhardness

For enamel, the percentage of surface microhardness alteration after treatment (%SMH_alt_) showed no significant differences (p=0.4894) among the groups (Table 2). For dentin, there were significant differences (p<0.0001) among the treatments, and Tukey’s test revealed that Si/PO4/MFP and Si/PO4/MFP/BS resulted in significantly increased microhardness compared to non-fluoride control toothpaste (Table 3).

**Table 2:** Mean percentage (Standard deviation) of microhardness data and results of Tukey test for enamel

Treatments	Surface Microhardness						% SMHalt = (SMHT / SMHE) X 100		
	B		E		T				

Control	361.02	± 15.07	278.29	± 29.47	292.58	± 9.36	106.0	± 9.3	A
Arg/Ca/MFP	351.31	± 14.40	257.08	± 11.88	284.15	± 11.44	110.8	± 8.2	A
Si/PO_4_/MFP	347.43	± 13.35	268.71	± 11.20	283.40	± 12.46	105.6	± 6.2	A
Si/PO_4_/MFP/BS	341.80	± 10.02	270.30	± 17.29	284.71	± 24.41	105.7	± 10.9	A

Uppercase letters show differences within treatments

**Table 3:** Mean percentage (Standard deviation) of microhardness data and results of Tukey test for dentin

Treaments	Surface Microhardness						% SMHalt = (SMHT / SMHE) X 100		
	B		E		T				

Control	63.44	± 4.69	14.68	± 1.30	14.64	± 1.05	100.5	± 11.80	A
Arg/Ca/MFP	62.62	± 2.17	14.84	± 2.95	17.00	± 2.82	116.2	± 17.13	AB
Si/PO_4_/MFP	62.84	± 4.44	15.07	± 3.07	19.50	± 3.59	132.4	± 30.13	BC
Si/PO_4_/MFP/BS	63.85	± 4.06	14.3	± 1.12	22.88	± 4.59	161.1	± 35.18	C

Uppercase letters show differences within treatments

### Profilometry

Profilometric analysis was performed after the 3^rd^ and 5^th^ days of the cycle to assess surface loss. RM ANOVA test showed differences among the dentifrices for enamel and for dentin both after 3 and 5 days. For enamel, Tukey’s test showed that, for 3 days, all groups were similar to the control, and Si/PO_4_/MFP, with and without the serum, presented higher surface loss than Arg/Ca. For 5 days, all groups presented surface loss values similar to the control. Regarding time, all groups presented increase in surface loss, except Si/PO_4_/MFP/BS, which maintained similar values.

For dentin, after 3 days of cycling, only Si/PO_4_/MFP/BS presented lower values of surface loss compared to the other dentifrices tested (Table 4). With the maintenance of the erosive-abrasive cycle, after 5 days, the application of the boost serum in the Si/PO_4_/MFP/BS group maintained the lower dentin loss, but it was not significantly different from the control group. Regarding time, all groups presented similar values of surface loss, except Arg/Ca, which presented higher loss. Table 4 shows the mean values obtained for enamel and dentin surface loss.

**Table 4:** Mean (Standard deviation) of surface loss and results of Tukey test for enamel and dentin after 3 and 5 days of the erosive/abrasive cycling (in mm)

Dentifrices tested	Enamel						Dentin					
	3 days			5 days			3 days			5 days		
	Mean	SD		Mean	SD		Mean	SD		Mean	SD	

Control	2.21	±0.58	ABa	2.91	±0.65	Ab	3.90	±1.09	Aa	4.09	±1.01	ABa
Arg/Ca/MFP	1.82	±0.50	Aa	2.87	±0.57	Ab	4.04	±0.82	Aa	5.02	±0.85	Cb
Si/PO_4_/MFP	3.09	±1.22	Ba	3.82	±0.82	Ab	4.57	±0.44	Aa	4.66	±0.59	BCa
Si/PO_4_/MFP/BS	2.98	±0.96	Ba	3.49	±0.87	Aa	2.76	±0.64	Ba	3.26	±0.24	Aa

Uppercase letters show differences between dentifrices for each substrate (enamel or dentin). Lowercase letters show difference between time (3 x 5 days).

### Abrasivity analysis

**Figure 2:**
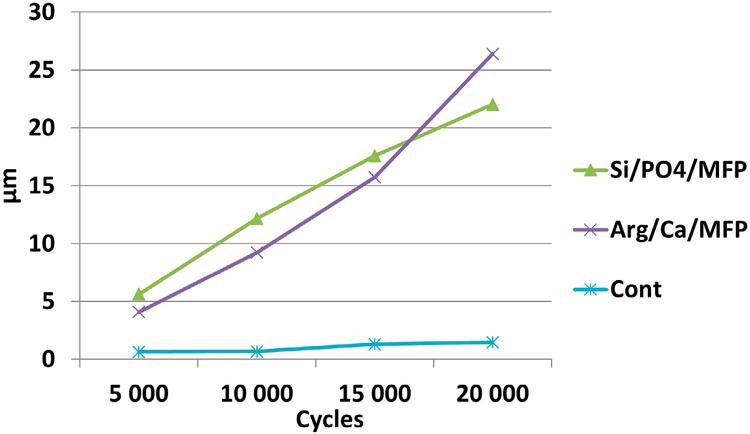
Means of dentin surface loss after 5,000 (1), 10,000 (2), 15,000 (3) and 20,000 (4) abrasive cycles

Dentin abrasiveness pattern was measured quantitatively by profilometry. One-way ANOVA showed that after 20,000 cycles the control dentifrice, without fluoride, was the less abrasive one. Arg/Ca/MFP and Si/PO_4_/MFP presented similar intermediate abrasivity potential. The graph at Figure 2 shows the surface loss after 5,000, 10,000, 15,000 and 20,000 abrasive cycles. There was no significant correlation between enamel and dentin surface loss at the end of the cycling and dentin abrasivity after 20,000 abrasive cycles (r enamel loss × dentin abrasivity = 0.87; r dentin loss × dentin abrasivity = 0.57; all p>0.05).

## Discussion

Toothpastes play an important role in oral hygiene, since they are affordable, easy to obtain, and have been traditionally incorporated into the dental hygiene routine of individuals. Many products offer multiple benefits due to the addition different active ingredients.^26^ Therefore, the toothpaste stands out as an interesting vehicle for providing agents to control ETW^26,27^ and its undesirable consequences, such as tooth sensitivity. The products tested in this study exhibited no significant differences on eroded enamel microhardness and enamel loss, thus the tested null hypotheses were rejected for enamel substrate. For dentin, the null hypotheses were accepted, since significant differences were observed for the different treatments.

Our study was conducted using an erosion-abrasion *in vitro* model, evaluating the behavior of the toothpastes in the distinct phases of the erosive process. In the first day of the cycle, the microhardness of the substrates was measured, and the non-fluoride control toothpaste resulted in lower dentin microhardness values compared to the Si/PO_4_/MFP products. However, the profilometric results showed similar surface loss between the control toothpaste and the other groups. Considering that the control did not contain fluoride in its formula, higher values of surface loss would be expected. This may be related to the low abrasiveness of the control toothpaste (as shown in Figure 2), which promoted reduced surface loss, matching the effect of the fluoride present in the other toothpastes, thus showing higher abrasiveness.

The results of our study indicate that the toothbrush abrasion played an important role on the efficacy of the products tested, by modulating the benefits given by their chemical active agents. However, we found no correlation between the surface loss and the abrasiveness of the dentifrices for enamel or dentin. Although the surface profile comparison can be used as an alternative method to measure dentifrice abrasiveness, its accuracy in differentiate among categories is not as effective as the Relative Dentin Abrasivity (RDA) method.^28^ Since standardization of the RDA values is only possible with experimental toothpastes, the comparison among commercial toothpastes is challenging due to the complexity of their active ingredients and abrasivity potential.^21,29^

Regarding the active ingredients, previous studies have shown that the presence of fluoride can offer some protection for eroded tooth tissues,^8,25,30^ but this beneficial effect is dependent upon dosage and type, meaning that not all fluoride dentifrices are equally effective.^7,26^ All the toothpastes tested in this study present similar concentration of fluoride (1450 ppm) as monofluorphosphate (MFP), which does not allow an optimized fluoride release under *in vitr*o conditions, since it requires to be broken down by salivary proteins.^31,32^ Thus, a higher fluoride availability would occur under *in vivo* conditions. Still, the calcium and phosphate contents of the artificial saliva and of the formulas of the toothpastes could have reacted with the fluoride during the slurry preparation, also decreasing the availability of the free fluoride released.^11^

Regarding the Si/PO_4_/MFP toothpaste, *in vitro* and *in situ* studies showed that its formulation based on calcium silicate and sodium phosphate salts (monosodium phosphate and trisodium phosphate), and MFP presented efficacy against enamel demineralization and was also able to improve its rehardening^14,15,21^. The presence of calcium silicate is expected to release calcium ions into the oral fluids under erosive conditions, increasing their saturation, thus reducing enamel dissolution.^16,33^ Furthermore, calcium silicate may act as a chemical and physical barrier against acids due to its ability to cause pH buffering and the formation of a hydroxyapatite-nucleated layer.^16,33^ Our results showed that the Si/PO_4_/MFP toothpaste alone was not able to promote higher values of enamel microhardness after treatments compared to the control toothpaste. Moreover, the system did not significantly protect the enamel against advanced tissue loss. This suggests that the phosphate and calcium-based salts, that promotes the deposition of calcium silicate particles onto the softened enamel, were not able to effectively resist the toothbrush abrasion.^18^ The favorable results reported previously with this toothpaste^14,15,20^ are usually related to its protective effect against acid challenges, since abrasion was not considered in many studies. The presence of abrasion modulates the process and increases the complexity of choosing a control group. A previous study showed favorable results when Si/PO_4_/MFP toothpaste was compared to experimental products with similar composition and abrasiveness potential.^21^

When applied to dentin, the Si/PO_4_/MFP toothpaste associated or not to the boost serum was effective to increase microhardness of the previously demineralized substrate when compared with the control, but the toothpaste, without the serum, presented limited efficacy in preventing dentin erosion under abrasive conditions, as reported previously.^12^ However, the application of the boost serum resulted in the reduced dentin surface loss under erosive/abrasive challenges compared to the use of toothpaste only. The greater fluoride availability, due to the additional presence of sodium fluoride in the serum, and the longer contact time are thought to be the main responsible for the enhanced protection of the association between the toothpaste and the dual-phase gel, especially on dentin. The tubular morphology and demineralized organic layer present on the eroded dentin surface may favor both fluoride and calcium retention.^34^ The application of the boost serum for three days followed the manufacturer recommendation, therefore profilometry was assessed after the 3^rd^ day, to verify its immediate protective effect, and after five days, to quantify the evolution of tissue loss promoted by different treatments. For dentin, the highest protection against erosive wear was obtained for Si/PO_4_/MFP/BS group with three days. However, this improved efficacy provided by the serum was not significantly different from the control group after 5 days, although it promoted lower dentin loss than the Si/PO_4_/MFP and Arg/Ca/MFP toothpastes, this might suggest the necessity of regular application of the serum for a sustained effect.

The presence of arginine in Arg/Ca/MFP did not show improved efficacy on protecting the enamel and dentin against erosion. Arginine and calcium carbonate acts by deposition, physically sealing the exposed dentin tubules and forming a mass composed by calcium, phosphate and arginine that reduces acid solubility.^35^ Although this dentifrice has shown the ability to reharden enamel softened by an previously erosive challenge,^36^ the results from this study do not indicate a superiority compared to the other formulations tested. The presence of arginine and calcium carbonate did not, comparatively, improve the protection against erosive wear neither for enamel nor for dentin, corroborating Ervesole, et al.^37^ (2014). The dentin tubule occlusion promoted by arginine is not able to withstand the initial erosion or frequent acid challenges.^38-41^

The complex composition of the toothpaste formulations difficult the comparison when they are tested under erosion or erosion-abrasion. Besides their abrasiveness (composition, size, and distribution of particles), the excipients, including thickening agents, surfactants and viscosity, may modulate the protective effect of their active agents.^22,29,42^ Thus, the choice of an adequate standard control toothpaste is a hard task. Moreover, the extrapolation of the results of this *in vitro* study to the clinical situation must be carefully performed, since the action of saliva composition, clearance, and acquired pellicle were not considered.

## Conclusions

The calcium silicate/sodium phosphate/fluoride toothpaste associated to the boost serum showed favorable effect on dentin microhardness, however this was not maintained with the persistence of the erosive-abrasive challenges. Similar enamel and dentin loss was observed when this system was compared to the non-fluoride control toothpaste. The abrasivity potential of the toothpastes could not predict their effect on erosive tooth wear.
